# *In Vivo* Hypolipidemic, Hypoglycemic, Antihyperglycemic, and *In Vitro* Antioxidant Effects of *Podocarpus gracilis* Leaf Extract and Fractions in Diabetic Mice

**DOI:** 10.1155/2023/9187837

**Published:** 2023-10-06

**Authors:** Wakuma Wakene Jifar, Ahmed S. BaHammam, Yadeta Babu Bayane, Biruk Tafese Moges, Teshome Bekana

**Affiliations:** ^1^Department of Pharmacy, College of Health Sciences, Mattu University, Mettu, Ethiopia; ^2^University Sleep Disorders Center, College of Medicine, King Saud University, Riyadh, Saudi Arabia; ^3^Strategic Technologies Program of the National Plan for Science and Technology and Innovation in the Kingdom of Saudi Arabia, Riyadh, Saudi Arabia; ^4^Department of Medical Laboratory Sciences, College Health Sciences, Mattu University, Mettu, Ethiopia

## Abstract

**Background:**

*Podocarpus gracilis* is an evergreen, dioecious tree found in Ethiopia and other African nations. It can reach a height of 60 meters. Without any scientific validation, ethnobotanical studies conducted in Ethiopia revealed that the *Podocarpus gracilis* plant's leaf is consumed orally to treat diabetes mellitus. Hence, this study aims to evaluate the *in vivo* blood glucose level lowering, lipid-lowering, and *in vitro*-free radical scavenging responses of *Podocarpus gracili*s leaf extract and fractions on experimental mice induced with diabetes.

**Methods:**

The *in vitro* antioxidant activity of PGC (*Podocarpus gracilis*) leaf extract was assessed by using a diphenyl-2-picrylhydrazyl (DPPH) assay. The oral glucose-loaded, normoglycemic, and streptozotocin- (STZ-) induced diabetic mouse models were employed. In the STZ-induced mice model, the leaf extract and solvent fractions activity on serum lipid and weight were also measured. The extract and fractions were tested at 100, 200, and 400 mg/kg dosages. One-way ANOVA was used to determine the statistical significance of BGL (blood glucose level) changes within and between groups, and Tukey's post hoc multiple comparisons were then performed.

**Results:**

In the acute toxicity study of *Podocarpus gracilis* leaf extract and fractions, there was no evidence of animal mortality at the maximum dose of 2 g/kg during the observation period. The extract-treated group with normoglycemia revealed a significant lowering in BGL at the 4-hour mark of 27.4% (*p* < 0.001) and 25.2% (*p* < 0.01) at doses of 200 mg/kg and 400 mg/kg, respectively, compared to that in negative control. In the oral glucose tolerance test (OGTT) model, only 400 mg/kg treated groups at 120 min after exposure showed a BGL reduction of 31.17% which was statistically significant (*p* < 0.05) in comparison to the negative control. In the single-dose STZ-induced model, eighth-hour BGL measurements from CE 100, CE 200, CE 400, and GLC5 showed drops in BGL of 43.1%, 44.1%, 45%, and 47.3% from baseline fasting BGL values. In the repeated streptozotocin (STZ)-induced model, at all doses of leaf extract and fractions, the fasting BGL was significantly (*p* < 0.001) reduced. Moreover, the leaf extract and solvent fractions have shown a significant (*p* < 0.001) reduction of serum lipids such as LDL, TC, and VLDL, and at the same time, it increases HDL at 14 days with body weight gained. In the test for antioxidant activity, the half-maximal inhibitory concentrations (IC_50_) for leaf extract and the standard medication (ascorbic acid) were 8.2 *μ*g/ml and 3.3 *μ*g/ml, respectively. The IC_50_ value denotes the concentration of the sample required to scavenge 50% DPPH radicals.

**Conclusion:**

The 80% hydromethanolic leaf extract and fractions of *Podocarpus gracilis* exhibited blood glucose lowering, lipid-lowering activity in normoglycemic, oral glucose tolerance test (OGTT) mode, and STZ-induced diabetic mice with weight gains. There is scientific support for the alleged traditional use as an antidiabetic, lipid-lowering, and antioxidant activity. The results need to be confirmed by future studies.

## 1. Introduction

### 1.1. Background

Insulin secretion, action, or a combination of both is compromised in diabetes mellitus (DM), a metabolic disorder. Today, both children and adults are affected by this chronic condition [1, 2]. A report from an international diabetic federation (IDF) revealed that there were around 463 million diabetics worldwide in 2019, and by 2030 and 2045, those numbers will rise to 578 and 700 million, respectively. The major form of diabetes, type 2 diabetes (T2DM) is a rapidly increasing global phenomenon [3]. It is a leading source of problems such as CVD, retinopathy, neuropathy, nephropathy, and infertility that cause patients and their families to experience severe emotional and physical distress [4]. Because it is linked to increased levels of circulating free fatty acids, cytokines, and other mediators of cellular damage that promote insulin resistance and impair beta-cell function on the pathway to diabetes development, obesity is one of the main risk factors for type 2 diabetes. It also increases the cardiovascular complications of DM [5]. Diabetes and its consequences are still difficult to cure despite the development of numerous antihyperglycemic medications from both natural and synthetic sources [6]. Research interest in finding more potent and secure antihyperglycemic medications to add to the therapeutic options has continued not just over the past few decades but also in the present [7].

WHO advises that novel antidiabetic medications come from medicinal plants that are both secure and efficient at treating diabetes mellitus [8]. Drugs for treating human illnesses have always come from plants, and also, in many parts of the world, especially in the rural areas of developing countries, since conventional drug options do not exist necessitating reliance on traditional plant treatments to enhance their main healthcare, source of income, and standard of living [9, 10]. There are approximately 1200 plants with antidiabetic qualities, indicating a high need for research on chemicals derived from plants to create novel antidiabetic drugs [11]. Numerous clinical studies have demonstrated that plants have potent antidiabetic effects on people [12]. Therefore, therapeutic alternatives should contain antihyperglycemic activities with long-term safety-proven antihyperlipidemic and antioxidant effects for coexisting diabetes and metabolic diseases [13]. Current diabetes treatments work by boosting pancreatic insulin production, among other glycemic control strategies [14], decreasing intestine absorption, increasing insulin receptor sensitivity, and reducing hepatic gluconeogenesis. However, none of these treatments can cure diabetes, and the majority of them are pricy and frequently have side effects [15]. The use of plant-derived bioactive compounds, which are effective, accessible, safe, and less expensive, is thus a therapeutic option for DM [9]. Numerous animal models of the well-known diabetogenic drug streptozotocin (STZ) (N-methyl nitro carbamoyl-D-glucosamine) have been used as screening models to assess the antidiabetic properties of herbal remedies [16]. This chemical effectively methylates DNA and supplies nitric oxide to pancreatic cells. Due to the low levels of free radical scavenging enzymes in pancreatic cells, these cells are particularly vulnerable to nitric oxide (through loss of aconitase function) and free radical damage. STZ causes symptoms resembling those of diabetes by destroying insulin-secreting pancreatic beta-cells and decreasing insulin production [17]. The cause of many diseases, including diabetes mellitus and its consequences, results in the induction of oxidative stress. According to several studies, antioxidants help treat diabetes and its side effects by lowering oxidative stress [18].

*Podocarpus gracilis* is a green plant that can reach 60 meters, which is available in Ethiopia and known among locals as Birbirsa in Afaan Oromo, Zigiba in Amharic, and Podo or East African Yellow Wood in trade, and it is also found throughout Kenya, Tanzania, Mozambique, South Africa, and Madagascar [19]. In the ethnopharmacological survey, *Podocarpus gracilis* was shown to have substantial therapeutic benefits, including a root for treating an evil spirit (being possessed by demons) is one of the oldest ways of accounting for bodily and mental disorders, leaves for treating rabies and hair loss, and roots and leaves for treating bone fractures [20]. According to a study carried out in southwest Ethiopian, after the COVID-19 pandemic crisis, there was a low supply of essential drugs used for the treatment of noncommunicable diseases like diabetes mellitus, and due to that reason, the community the surrounding started to utilize medicinal plants (94.9%) without safety and efficacy validation [21, 22]. One of the most utilized medicinal plants is *Podocarpus gracilis* plant to treat diabetes [23, 24]. However, the effects of the crude leaf extract on BGL have not been examined scientifically. A pharmacological study has revealed that plants in the *Podocarpaceae* family exhibit potent insulin-mimetic and antidiabetic properties [25]. Lactones, phytoecdysteroids, and flavonoids are only a few of the chemical, biological, and pharmaceutically intriguing substances that the genus *Podocarpus* is known to produce [26], which, as seen in previously published studies, can enhance insulin secretion by inhibiting DPP-IV, so preventing the active versions of the incretin hormones, GIP (1–42) and GLP-1, from degrading, and which have also been shown to have this capacity [27].

One of the monotypic genera in the *Podocarpaceae* family is *Podocarpus gracilis* [28]. So, it is possible that the research plant shares antidiabetic properties with another member of this family of plants. In addition, cytotoxic drugs are therapeutically advised for their antidiabetic effects [21, 22]. Presence or absence of different compounds can be used to relate the new and the old taxa of *Podocarpaceae* family. For example, flavonoids can be a useful chemotaxonomic tool in this group of plants, since bioflavonoids of the amentoflavone and hinokiflavone groups have been shown to be good taxonomic markers in the great majority of this genus [29]. Following these, *Podocarpus gracilis'* bioactive components have been shown to have cytotoxic and antioxidant effects *in vitro* [30]. Important essential oils such as cineole, citronellal, and citronellic acid are also known to be present [31]. Essential oils also have potential antioxidants [32]. Podolide (antileukemic norditerpene and dilactone) is the first compound of this class of family reported to show a tumor-inhibitory activity. Similarly, this compound was responsible for the tumor-inhibitory activity of an ethanol extract of the twigs and leaves of *P. gracilior* [31]. The preliminary structure-activity relationship studies suggested the methoxyl and hydroxyl groups in bioflavonoids and monoflavonoids, respectively, play a crucial role in mediating cytotoxic activity [33]. Since these compounds were identified in *P. gracilior*, it has been verified as it has cytotoxicity effect. A species of Podocarpus has been reported to be resistant to many insects, and nor- and bis-norditerpenes such as nagilactones from these plants have been shown to be responsible [29].

Compound such as nagilactone C, D, and F, isolated from *P. gracilior* showed an insect-feeding-deterrent activity and an insecticidal activity [34, 35]. Norditerpenes and totarols from Podocarpus are known to have cytotoxic activities against several forms of cancer including, P388 murine leukemia cells. The same result in this experimental study, Taxol, isolated from *P. gracilior* Pilger inhibits the growth of HeLa cells (human cancer cells) and is a promising new treatment for several forms of cancer [28].

The experimental plant may have a reduction in BGL because of its confirmed antioxidant activity. This is because an imbalance between the generation of free radicals and their antioxidant qualities can lead to the appearance of numerous pathological disorders, including diabetes. The effects of *Podocarpus gracilis* leaf extract and solvent fractions on hypolipidemic, hypoglycemic, antihyperglycemic, and *in vitro* antioxidant activities in diabetic mice have not been scientifically examined. We hypothesized that *Podocarpus gracilis* leaf extract and solvents fractions have beneficial effects on BGL, serum lipid level, and antioxidant activities. Hence, this study aims to evaluate the *in vivo* blood glucose level lowering, serum lipid lowering, and *in vitro* free radical scavenging responses of *Podocarpus gracilis* leaf extract and solvents fractions on experimental mice induced with diabetes.

## 2. Methods and Materials

### 2.1. Chemicals and Instruments

Methanol was provided by Nice Chemicals, India; sodium citrate was provided by Lab Tech Chemicals; DPPH was provided by Sigma-Aldrich, Germany, and solution of 5% glucose was provided by Reyoung pharmaceuticals, China; Whatman filter paper No.1 and pipette. Beckman Colter, Germany-based automated chemistry analyzer; Agilent Technologies, Malaysia-based UV spectrophotometer; Hamato, Japan-based rotary evaporator; Tween-80 was obtained from Avishkar Lab Tech Chemicals in India; streptozotocin was obtained from produced by Fisco Research Laboratories in India; glibenclamide was obtained from Julphar Pharmaceuticals in Ethiopia; ascorbic acid was obtained from Blulux Laboratories in India; and Alliance International in Taiwan (Labfreez, China).

### 2.2. Plant Material

Fresh *Podocarpus gracilis (Podocarpaceae)* leaves were collected in March 2021 from the Yayu coffee forest, a town 50 kilometers from Mettu town which is the capital city of Illu-abba bor zone (located in the southwest of Oromia regional state, Ethiopian). After the plant material had been identified and authenticated by botanists, a voucher specimen (003WWJ/2021) was kept at Mattu University's biology departments for future use.

#### 2.2.1. Preparation of Plant Extract and Fractionations

*(1) Preparation of Plant Extract*. After being harvested, the plant's leaves underwent thorough cleaning with distilled water to eliminate dirt before being allowed to air dry at room temperature (25–27°C) with adequate ventilation. The leaves were electronically ground into a coarse powder, macerated in 80% methanol for 72 hours, and then filtered using Whatman No. 1 filter paper. The residue was macerated twice for intervals of 72 hours each using a fresh 80% hydromethanolic solvent. The filtrates from these macerations were subsequently concentrated under decreased pressure using two devices: a hot air oven (Medit-Medizin Technik, Germany) and a rotary evaporator (Hamato, Japan), both set to 40°C. It was dried, frozen in the refrigerator for a full night, and then lyophilized (Labfreez, China). The desiccator was then employed to keep the dried leaf extract until the experiment use. A total of 140 grams of dried *Podocarpus gracilis* leaf crude extract were obtained, with a yield of 17.4% (w/w) [36].

*(2) Fractionations*. Fractionation of leaf extract was carried out using water, ethyl acetate, and hexane solvents. Briefly, the leaf extract was dissolved in 400 mL of distilled water and this solution was transferred to a separating funnel. An equal volume of hexane was added to it and was shaken vigorously. The mixture was separated into two layers, waited for a while, and then, the hexane fraction was removed. The partition with hexane was repeated two times. The hexane layer was combined and subjected to evaporation using a hot air oven set at 40°C to get the hexane fraction. To the separating funnel containing the aqueous layer, 400 mL of ethyl acetate was added. The mixture was separated into two layers, and then, the ethyl acetate was separated and the procedure was repeated two times. The ethyl acetate layer was pooled and concentrated using a hot air oven set at 40°C to obtain the ethyl acetate fraction. The remaining aqueous layer was concentrated using a hot air oven set at 40°C and frozen in the refrigerator overnight and then, concentrated in a lyophilizer to remove the water. The % yield of the dried fractions was calculated and the fractions obtained were put in airtight bottles and stored in a refrigerator at −4°C until used [36].

### 2.3. Blood Glucose Level Measurement

In all animal models, blood samples were taken aseptically by cutting the tip of the tail of each fasted mouse [9]. A glucometer and test strips were used to determine the blood glucose level. The authors focused on blood glucose levels because blood glucose concentrations are considered an accurate diagnostic tool for diabetes and other parameters like lipid serum profile, weight, and free radical scavenging effect [37].

### 2.4. Experimental Animals

A total of 114 Swiss albino mice were used in the study (five female mice for acute oral toxicity study) because female mice are more sensitive than males. Estradiol's capacity to prevent pancreatic cells from apoptosis brought on by oxidative stress may be responsible for the lower sensitivity that females experience [38]. In addition, literature reviews of standard LD50 testing revealed that, while there are occasionally instances when variations are seen between the sexes, generally speaking, females are slightly more sensitive [39]. For the diabetic mice model, all mice employed were male because they are more sensitive to STZ and insulin than female mice [40]. They were distributed as follows, 30 male mice for the hypoglycemic model, 30 male mice for OGTT, and 84 male mice for the induced mice model. The number of mice was equal for each group (six mice per group). This was accomplished following prior similar study methods using diabetic mouse models [41].

Albino mice were purchased from the Ethiopian Public Health Institute (EPHI), located in Finfinne, the capital city of the Oromia regional state, Ethiopia. They were 6–10 weeks old, weighed 20–30 g, and the mice were housed in polypropylene cages with unlimited access to a pellet diet and water at all times in standard conditions (12-hour light/dark cycle, room temperature). Before the trial, the mice got accustomed to the lab setting for two weeks. The Research and Ethical Committee gave the Phar111/120/21 permission number at Mattu University's pharmacy department as their approval for the project.

All models were conducted by the eighth edition of the National Academy of Sciences Guide for the Care and Use of Laboratory Animals, published by the National Academy of Sciences, Institute for Laboratory Animal Research, Division on Earth and Life Studies, Washington, DC, USA [42].

### 2.5. Leaf Extract and Fractions' Phytochemical Screening

To gather preliminary information on the components of the plant extracts and solvent fractions, phytochemical screening was carried out utilizing color forming and precipitating chemical reagents on the dried leaves extract and solvent fractions of *Podocarpus gracilis*. Secondary metabolites, such as alkaloids, steroidal substances, phenolic substances, tannin, saponins, terpenes, and flavonoids, were examined for presence or absence in *Podocarpus gracilis* leaf extract and solvent fractions using standard techniques [43]. The specific methods that were employed to show the presence or absence of metabolites in all fractions and leaf extract are described in detail in Appendix.

### 2.6. Acute Oral Toxicity Test

An acute oral toxicity test for a crude extract of *Podocarpus gracilis* leaves and fractions was performed using the Organisation for Economic Cooperation and Development (OECD) No. 425 Guideline limit test guidelines. On the first day of the experiment, one female Swiss albino mouse received oral administration of 2 g/kg of the extract and solvent fractions. The mouse was watched for one day for any physical or behavioral abnormalities. Since the first mouse showed no evidence of toxicity, the remaining four female mice were enlisted the next day and starved for four hours. Then, mice were given a single oral dose of the 2 g/kg extract and fractions while being observed for any toxicity. For a total of two weeks, the observation was continued for any indication of toxicity [44]. Finally, the crude extract and fractions of the leaf of *Podocarpus gracilis* at 2000 mg/kg dose did not show any sign of toxicity or death in the 14-day follow-up period. Because, no visible adverse effects such as changes in feeding, body weight, hair erection, urination, lacrimation, salivation, and movement were observed, indicating the extract is safe.

### 2.7. Grouping and Dose Provided to Mice for Evaluation of the Effect of the Leaves Extract of *Podocarpus gracilis* on Blood Glucose Level

Because male mice are more susceptible to STZ and insulin than female mice, they were used in all *in vivo* experimental studies (normoglycemic, oral glucose loading, and STZ-induced diabetes animal models) [29–31] except in the case of acute oral toxicity studies. Furthermore, the experimental design described by the method in the previously studies was employed in grouping the mice [45]. In all models, the animal groups and the substance administered were as follows: common negative and positive controls were used for both the crude leaf extract and fractional tests. Group I, the negative control, was given distilled water (DW10 ml/kg), group II, the positive control, was given glibenclamide 5 mg/kg (5 mg/kg GLC), and groups III–V were given 100, 200, and 400 mg/kg of the crude leaf of 80% methanolic extracts (CE100 mg, CE200 mg, and CE400 mg) while groups III–XII were given fractional extracts at 100, 200, and 400 mg/kg of hexane fraction (HE100 mg, HE200 mg, and HE 400 mg), the ethyl acetate fraction (EF100 mg, EF200 mg, and EF 400 mg), and aqueous fraction (AF100 mg, AF200 mg, and AF 400 mg). Based on past studies, the standard medication was chosen to be glibenclamide [32]. Because the population consumes plant leaves orally, this oral administration approach was used for the study [46].

### 2.8. Induction of Experimental Diabetes

Streptozotocin (STZ) diabetes can be induced either by a single high-dose injection or by multiple low-dose injections [47]. We have used a single high-dose injection of STZ (150 mg/kg) because multiple low-dose injections may lead to insulitis and the slow destruction of the islets. A single large dose of STZ is used for experiments because, unlike the low dose of STZ, large doses can cause near-total destruction of beta cells and little or no measurable insulin production [48]. Moreover, a previous study found that STZ treatment (i.p.) at 150 mg/kg efficiently produced DM in physiologically normal mice, as evidenced by hyperglycemia [45]. In addition to that, STZ is most commonly delivered by one of the two routes, intraperitoneal (IP) or intravenous (IV). We preferred the intraperitoneal (IP) route because it is a quick and easy method of administration [49]. Moreover, an incomplete IV dose may cause variability in the onset or severity of diabetes, especially when injecting highly concentrated solutions. 30–40% mortality happened in animals induced with STZ because the most common adverse effect of the administration is hypoglycemic shock, leading to death [38]. In our experiment, we avoided this complication by giving 5% glucose solution following this procedure: after 6 hours of induction with STZ, mice were kept on 1 ml of 5% glucose solution to prevent hypoglycemia induced by STZ in the early course of its action. Three days (72 hours) after induction, each mice's plasma blood glucose level was determined, and animals with a fasting blood glucose level above 200 mg/dl were included in the study. Only mice with BGL less than 200 mg/dl were excluded from the study. Since the DM induction rate of streptozocin is not 100% in antidiabetic activity [50]. Unlike a chronic condition or diabetic complications in which the probability of mice death is 30–40% if not well managed, in this study, the length of the experiment was two weeks, so there was less chance of mice death [51, 52].

### 2.9. Effect of Leaf Extract of *Podocarpus gracilis* on Blood Glucose Level of Normal Fasted Mice

For 4–6 hours, Swiss albino male mice fasted, although they were free to consume water. This was carried out to determine how the crude extract affected the BGL in mice that were not diabetic. Then, five groups of six fasted mice were created, and each group received its predetermined treatment. Blood samples were taken from mice immediately before treatment (at 0 hours) as a baseline, and then at 1-, 2-, 3-, and 4 hours after treatment, and blood glucose level (BGL) was then determined in these samples [53].

### 2.10. Effect of Leaf Extract of *Podocarpus gracilis* on Postprandial Glycemia in Nondiabetic Mice

To determine how crude extract might affect the OGTT in this model, the baseline BGL was obtained right before the medications were administered. After that, the mice were administered 2 g/kg of glucose solution orally 30 minutes after receiving the extract. Each animal's BGL was assessed at zero minutes before treatment and once more at 30, 60, and 120 minutes after receiving the extract [54].

### 2.11. The Hypolipidemic and Antihyperglycemic Activities of the Leaf Extract and Fractions of Plant Materials on Streptozocin-Induced Mice

In STZ-induced single diabetes mice, diabetic mice fasted for 14 hours before being randomly assigned to one of five groups (*n* = 6). Depending on the group the mice belonged to, they were given distilled water (DW 10 ML/kg), glibenclamide (GLC5mg/kg), and 80% hydromethanolic leaf crude extract and solvent fractions at a dose of 100 mg/kg, 200 mg/kg, and 400 mg/kg. The BGL was then assessed at baseline immediately before therapy (at 0 hours) and at 2, 4, 6, and 8 hours later. In repeated dose studies, BGL was measured at 0 days, 7 days, and 14 days. At the termination of treatment, i.e., 14 days, animals were deprived of food overnight. The lipid parameters such as total cholesterol (TC), triglycerides (TG), low-density lipoprotein (LDL), very low-density lipoprotein (VLDL), and high-density lipoprotein (HDL) cholesterol were evaluated using an automated chemistry analyzer [55]. After the experiment, mice were sacrificed according to the standard protocol of Drug Discovery and Evaluation: pharmacological assays by giving them a dose of (150 mg/kg) of sodium pentobarbitone intraperitoneally [55].

### 2.12. Antioxidant Activity of Leaf Extract of *Podocarpus gracilis* in Diphenyl-2-Picrylhydrazyl (DPPH) Assay

To assess the ability of the plant leaf extract to scavenge free radicals, we applied the technique outlined by Brand William et al. [56]. The leaf extract's potential antioxidant activity was evaluated based on its capacity to neutralize the stable DPPH free radical. A DPPH assay was developed (4 mg of DPPH and dissolved in absolute methanol of 100 ml, each time freshly prepared and stored in a dark and cool place). A 6 mg of ascorbic acid was dissolved in 3 ml of 100% methanol to produce an ascorbic acid solution (6000 g/3 ml, stock solution). 62.25, 125, 250, 500, 1000, and 2000 micro/ml dilutions of the stock solution were created [57]. The test sample solution, which was made up of 100 microliters of the plant's leaf extract and was dissolved in methanol at various concentrations (2000, 1000, 500, 250, 125, and 62.25 micron/mL), was then transferred to a different test tube that contained 3.9 mL of a 0.004% (w/v) DPPH radical solution, which was dissolved in methanol in separate test tubes. After 30 minutes, the absorbance at 517 nm was measured and recorded using a UV spectrophotometer. The same concentration of each sample was used for the test three times, and the inhibitory activity percentage was computed each time. The IC_50_ value denotes the concentration of the sample required to scavenge 50% DPPH radicals [58].

### 2.13. Statistical Analysis

For six mice in each group, all results were shown as the mean ± standard error of means (SEM). The statistical analysis was conducted using SPSS version 21.0, a statistical tool for social sciences. The results of a one-way ANOVA and Tukey's multiple comparison tests were used to compare the means of all parameters within each group as well as between groups. The result was considered significant at *p* < 0.05.

## 3. Results

### 3.1. Plant Extracts and Fractions

The 80% hydromethanolic extract of *Podocarpus gracilis* leaves was dried, and 17.4% of the dry matter was yielded as a percentage. From 140 grams (17.4% yields) of 80% hydromethanolic crude extract, and a yield of hexane, ethyl acetate, and aqueous fractions of the leaf of *Podocarpus gracilis* were 23.44%, 29.12%, and 40.23%, respectively.

### 3.2. Phytochemical Screening

Table 1 provides a summary of the test results that were obtained. Chemical analyses determined whether or not significant secondary metabolites such as saponins, phenolic compounds, and alkaloid chemicals were present. In all leaf extracts and fractions phytochemicals such as anthraquinones, glycosides, phenols, and flavonoids were identified. In contrast, terpenoids were identified only in crude extract. Low secondary metabolites were identified in the case of aqueous fractions.

### 3.3. Acute Toxicity Study

The *Podocarpus gracilis* leaf extracts and solvent fractions acute toxicity investigation did not indicate any animal deaths or any neurological, autonomic, physical, or behavioral toxicity indications at the upper dose limit of 2 g/kg during the observation period. Therefore, the leaf extract's LD_50_ is higher than 2 g/kg.

### 3.4. The Effect of Leaf Crude Extracts of *Podocarpus gracilis* on BGL of Normoglycemic Mice

Except at 0 hours and 4 hours after exposure, the overall change (i.e., lowering) of BGL was significant (*p* < 0.01) at all periods at a dose of 5 mg/kg GCL compared to the baseline BGL, a maximum reduction of 30.5% was observed at the fourth hour, with a highly significant (*p* < 0.001) reduction across all periods (0 hours). The greatest BGL reduction for the 200 mg/kg and 400 mg/kg PGC dose in this comparison was 27.4% (*p* < 0.001) and 25.2% (*p* < 0.01), respectively, at the 4-hour point. Similar comparisons were made in mice who received 100 mg/kg of the drug; none of the BGLs found at various time intervals were statistically significant when compared to the baseline BGL (0 hours), but the 4 hour BGL was considerably lower than the 1 hour BGL(*p* < 0.01). When the different doses of the extract-treated groups were compared with one another at all-time points, there was no statistically significant change in BGL (Table 2). Compared to the baseline, the greatest BGL reduction for those given 100 mg/kg extract was 16.6% in the fourth hour.

### 3.5. The Effects of the Leaf Extract of *Podocarpus gracilis* on Oral Glucose-Loaded Nondiabetic Mice

Before administering the vehicle, glibenclamide, and extract (*t* = 0 min), the BGL of all groups did not appear to vary from one another. However, all groups responded to the oral glucose load by showing an increase in BGL at 30 minutes (1 hour after oral glucose loading). When compared to the baseline, DW100 ml did not significantly reduce hyperglycemia in response to a glucose challenge at any time point.

At 120 minutes, the percentage reduction in BGL for extract doses of 100 mg/kg, 200 mg/kg, and 400 mg/kg compared to the baseline was 25.4, 28.6, and 31.17%, respectively. Mice receiving 100 mg/kg and 200 mg/kg did not differ significantly when their mean BGLs were compared within groups at various time intervals. In contrast, in the oral glucose tolerance test (OGTT) model, 400 mg/kg treated groups at 120 min after exposure showed a BGL reduction of 31.17% which was statistically significant (*p* < 0.05) in comparison to the negative control. Moreover, mice treated with glibenclamide demonstrated a statistically significant (*p* < 0.01) decrease in BGL at the 60^th^ and 120^th^ minutes of the post-treatment period than that of the negative control group (Table 3).

### 3.6. Single Dose Antihyperglycemic Effect of Leaf Extracts and Fractions in Diabetes Mice

Table 4 provides a summary of the effects of *Podocarpus gracilis* crude extract and fractions on the BGL of STZ-induced diabetic mice. All of the mice used for this model survived until the completion of the experiment. A statistical comparison of the groups revealed that DW10 ml/kg had no significant effect on BGL at the starting level at any point in time. To identify BGL differences, both within-group and between-group analyses were used. The baseline fasting BGL between the groups did not significantly differ, according to the between-group analysis. In addition, CE100 at the sixth and eighth hours (*p* < 0.001) and CE200, and CE400 at the fourth (*p* < 0.01), sixth (*p* < 0.001), and eighth (*p* < 0.001) hours both caused a significant reduction in BGL. Similar to this, except for the second and fourth hours following treatment with all ethyl fraction doses (100, 200, and 400 mg/kg) significantly (*p* < 0.001) decreased mice's BGL at all-time points compared to the negative control. Mice treated with aqueous fraction at a dose of 100 and 200 mg/kg showed no significant BGL reduction than that of the negative control group at all-time points. Moreover, the mice treated with GLC 5 mg/kg revealed significant (*p* < 0.05) BGL reduction for the second hour and (*p* < 0.001) for the other time points of post-treatment at all-time periods in comparison to the baseline level and the negative control. The HE 100, HE 200, HE 400, and GLC 5 mg/kg treatment groups displayed the highest percentage of reductions in BGL at 43.1%, 44.1%, 45%, and 47.3%, respectively, when compared to their respective baseline BGL levels at the 8^th^ hour.

### 3.7. Repeated Dose Antihyperglycemic Effect of Leaf Extracts and Fractions in Diabetes Mice

At day zero, BGL was significantly different in streptozotocin-induced mice than that of normal control mice (*p* < 0.001). When compared to diabetic control, the leaf extract, HE, and EF at doses of 200, 400, and GLC5 significantly (*p* < 0.001) reduced BGL on day 14. The BGL on the 7^th^ and 14^th^ days at all-time points did not decrease with the leaf extract, HE, or EF at a dose of 100 mg compared to the baseline blood glucose level (Table 5).

### 3.8. The Effect of Leaf Extract and Fractions of the Plants on the Body Weight of Mice

Before extract and fractions were administered (0 days), there was no significant difference between any of the groups' body weights and those of the normal control group. On the seventh day of treatment, significant body weight gains were observed for CE200 (*p* < 0.05), CE400 (*p* < 0.01), and GLC5 (*p* < 0.001) when compared to the diabetes control group. Moreover, on day 14 of treatment with solvent fractions of ethyl acetate and hexane at all doses (100, 200, and 400), body weight was significantly (*p* < 0.001) improved compared to diabetes control (Table 6).

### 3.9. The Effect of Leaf Extract and Fractions on the Lipid Profile of Diabetic Mice

The induction of diabetes dyslipidemia was confirmed by a substantial (*P* < 0.01) increase in serum total cholesterol and triglyceride levels and a significant decrease in HDL cholesterol in diabetic controls than that of the normal control (Table 7). When given to diabetic mice for 14 days, the leaf extract, HE, EF at (200 mg/kg and 400 mg/kg) dose, and GLC5 mg resulted in a substantial (*p* < 0.001) dose-dependent decrease in serum levels of TC, TG, LDL, and VLDL while increasing levels of HDL-c in comparison to the diabetic control group. In contrast, leaf extract and HE and EF at a dose of 100 mg/kg exhibited no significant decrease in serum levels of TG and LDL compared to baseline.

### 3.10. Antioxidant Activities

The standard medication for the DPPH method of measuring antioxidant activity was ascorbic acid. Using the DPPH free radical scavenging experiment, the antioxidant activity of the hydromethanolic leaf extract was investigated. This study demonstrated that the leaf extract's capacity to scavenge free radicals is concentration-dependent. The assay revealed that the leaf extract and ascorbic acid's IC_50_ values were 8.2 and 3.3 *μ*g/mL, respectively (Table 8). The IC_50_ value denotes the concentration of the sample required to scavenge 50% DPPH radicals.

## 4. Discussion

Diabetes mellitus (DM), a collection of metabolic illnesses, is the world's fastest-growing chronic disease and a danger to global public health [59]. High blood glucose levels are a symptom of diabetes mellitus (DM), which is caused by an absolute or relative lack of insulin production and/or insulin resistance. These high blood glucose levels can cause several further acute or chronic problems [60]. For many years, it has been known that medicinal plants and their phytochemicals have important pharmacological and biological advantages. As a result, their use as an alternative to synthetic medication therapy has increased [61]. Although *Podocarpus gracilis* has been used traditionally for treating diabetes mellitus in Ethiopian folk medicine [23], the *in vitro* radical scavenging and *in vivo* hypoglycemic, hypolipidemic, antihyperglycemic, response of *Podocarpus Gracilis* leaf extract, and fractions in Diabetic mice was not validated. Hence, this study aims to evaluate the *in vivo* blood glucose level lowering, lipid lowering, and *in vitro* free radical scavenging responses of *Podocarpus gracilis* leaf extract and solvent fractions on experimental mice induced with diabetes.

In the current study on acute oral toxicity (the method is easiest to apply to materials that produce death within one or two days; the method would not be practical to use when considerably delayed death (five days or more) can be expected) [62], the crude extract of the leaf of *Podocarpus gracilis* at 2000 mg/kg dose did not show any sign of toxicity or death in the 14-day follow-up period. Because, no visible adverse effects such as changes in feeding, body weight, hair erection, urination, lacrimation, salivation, and movement were observed, indicating the extract is safe. This finding showed that the plant's extract and fractions have an LD_50_ greater than 2 g/kg, proving that it is not harmful [63, 64].

In the normoglycemic model, the administration of *Podocarpus gracilis* leaf extract to normal mice at all evaluated doses (100, 200, and 400 mg/kg) demonstrated a minor hypoglycemic impact in comparison to the negative controls. However, glibenclamide-treated mice displayed a statistically significant decline in BGL when compared to the negative control group. This might be explained by the extract's inability to induce hypoglycemia in mice that had an intact and normal pancreas. It might also be because regular pancreatic function superdominates in regulating insulin secretion, which regulates glucose levels through physiological systems that act as counter-regulatory mechanisms [61, 65]. In contrast, glibenclamide's hypoglycemic impact was noticeable because it stimulated pancreatic cells to produce insulin and inhibited glucagon secretion [66]. The leaf extract may not be able to increase insulin release from beta cells, and its likely mode of action is distinct from that of glibenclamide 5 mg/kg, the standard medication used to treat DM. *Podocarpus gracilis* leaf extracts have qualities resembling those of well-known biguanide medications, particularly metformin, which, despite being an antihyperglycemic, has no effect on hypoglycemia in normal mice. Therefore, the putative mechanism of action of leaf extract may be comparable to that of metformin, which boosts insulin action, raises the number of glucose transporters, inhibits gluconeogenesis, lowers intestinal absorption, and encourages glucose utilization in the liver [67].

We followed established protocols for the oral glucose tolerance test (OGTT), which assesses the body's capacity to utilize glucose and detects diabetes mellitus [68]. The increase in BGL at the 30-minute mark of drug exposure during an OGTT validates the physiological induction of hyperglycemia brought on by oral glucose loading. In this test, the percentage reduction in BGL over the baseline was 25.4, 28.6, and 31.17% for extract doses of 100 mg/kg, 200 mg/kg, and 400 mg/kg at 120 minutes, respectively. Mice receiving 100 mg/kg and 200 mg/kg of PCG did not differ significantly when their mean BGLs were compared within groups at various time intervals. However, when compared to the starting period, 400 mg/kg treated groups at 120 minutes after exposure showed a BGL reduction of 31.17% which was statistically significant (*p* < 0.05) in comparison to the negative control. This could be because of the delayed beginning of the effect, as it has been seen in diabetic situations that the extract only begins to statistically significantly reduce hyperglycemia after two hours of treatment [69].

Moreover, according to the findings, it is also possible that there are at least two antihyperglycemic principles at play, one of which acts orally and suggests glibenclamide-type-type activity, and the other of which acts intravenously and strongly suggests insulin-type activity [70]. A well-known insulin secretagogue, glibenclamide, is effective in diabetes-induced mice. The other possible mechanism for leaf extract may be an insulin-like effect with a longer half-life, as reported for *Momordica charantia* [71] and *Cuminum nigrum* medicinal plants, or even a b-receptor antagonist [72]. Therefore, the plant's ability to tolerate oral glucose suggests that its leaves can help reduce diabetic complications connected to hyperglycemia having a similar mechanism with standard medication, which is insulin [73].

According to single dose antidiabetic study, the standard medication (GLC5mg/kg) significantly reduced BGL at the second (*p* < 0.05), fourth (*p* < 0.01), sixth (*p* < 0.01), and eighth (*p* < 0.001) hours in the STZ-induced DM in mouse models when compared to the negative control. In contrast to the normoglycemic model, at a dose of 100 mg/kg, the leaf extract(CE), hexane (HE) fraction, and ethyl acetate fraction (EF) had antidiabetic effects on mice receiving streptozocin, indicating that diabetes may alter the normal blood glucose regulating systems, making the lower dose's hypoglycemic properties visible [67]. The results also demonstrated that the extract's antidiabetic action grew over time, reaching its peak at the eighth hour (Table 8). Since other plants demonstrating antidiabetic properties followed a similar trend, the extract's active ingredients probably require time to concentrate sufficiently at the intended site [74]. According to a previous study, medicinal plants such as a *Ganoderma lucidum* extract contain phytochemicals such as flavonoids, phenolic compounds, alkaloids, terpenoids, saponins, tannins, glycosides, glycolipids, dietary fibers, carotenoids, and anthocyanins. These phytochemicals have potent antidiabetic effects by the mechanism of increasing insulin secretion and action, lowering gluconeogenesis, inhibiting glucosidase, and amylase enzymes, protecting pancreatic cells from oxidative stress and inflammation, increasing glucose transporter expression and translocation, promoting pancreatic cell proliferation, and preventing apoptosis [75]. Moreover, blood sugar-lowering effects of flavonoids and other polyphenol phytochemicals are primarily caused by increased pancreatic beta-cell GLUT-2 expression as well as increased GLUT-4 expression and translocation [76–78]. In the current study's preliminary qualitative phytochemical screening, the presence of phenols, flavonoids, tannins, alkaloids, glycosides, and steroids in the hydromethanolic leaf extract and solvents fractions of *Podocarpus gracilis* suggests that the plant extract's and fractions capacity to lower blood sugar levels may be caused by the presence of these phytochemicals, which may be the potential mechanism of action for the antidiabetic effect of leaf extract and fractions. Moreover, compared to the plant extract and the fractions, the standard medication had a relatively quicker beginning (at two hours) of the antidiabetic effect. However, the *Podocarpus gracilis* extract doses showed a variable commencement of the action, which most likely happened due to other principles interfering, especially at larger doses. Higher glycemic index ingredients, like reducing sugars, may release free glucose after digestion and may have the propensity to raise BGL after absorption [79], especially while using leaf extract for the first time. The presence of such an effect may delay the effectiveness of the plant extract and fractions [80], specifically when it is administered at higher doses, and may lead to higher quantities of this type of chemical. If such an impact materializes in response to the active compounds' hypoglycemic actions, the onset of the activity may be delayed [37, 81]. These may be the other possible justification for the delayed hypoglycemic effect of leaf extract and fractions of *Podocarpus gracilis* than that of the standard drug, glibenclamide. Compared to leaf extract and hexane fraction, less reduction of BGL was observed in the case of aqueous fraction. This may be due to the presence of less secondary phytochemicals in aqueous fraction and because of that it was not further investigated for their *in vivo* antihyperglycemic activity of repeated daily doses on diabetic mice.

In the repeated daily doses, leaf extract, HE, and EF at a dose of 200, 400 mg, and GL5 mg showed a significant (*p* < 0.001) BGL reduction on the 14^th^ day when compared to diabetic control. In contrast, the leaf extract, HE, and EF at a dose of 100 mg failed to show a reduction of BGL on the 7^th^ and 14^th^ days at all-time points compared to baseline BGL. In addition to conventional treatment concepts, administering adequate antioxidants can prevent or delay diabetic problems by restoring destructed beta-cells [25, 57]. The *in vitro* DPPH scavenging assay points to *Podocarpus gracilis's* antioxidant ability. The standard utilized was ascorbic acid, and the percentage of inhibition was discovered to be an IC_50_ of 3.3 *μ*g/ml. Table 8 displays specific antioxidant activity results in further detail. The antioxidant activity of *Podocarpus gracilis* extract was discovered to be dose-dependent. The antioxidant activity of the leaf extract correlated with the phenolic and flavonoid compounds, because a previous study showed plants having phenolic and flavonoid has antioxidants active due to free radical scavenging ability by giving hydrogen atoms to free radical chemical [82]. It is believed that the leaf's potent capacity to act as a donor of hydrogen atoms or electrons is what causes its remarkable capacity to scavenge free radicals [83]. The other possible reason for the antidiabetic activity of *Podocarpus gracilis* in repeated dose study for 14 days may be due to the antioxidant activity of the plant since it has been a validated antioxidant in the current investigation.

A key indicator of successful diabetes development in experimental mice is weight loss [84]. All mice displayed a drop in body weight three days after receiving an STZ injection [85]. In the current study, over 14 days, the diabetic group continuously decreased in body weight. A lack of insulin or irregularities in the catabolism of macronutrients like fat and protein could be to blame for the drop in body weight, which would result in significant tissue protein loss and muscle loss [86]. Hence, the weight gains after repeated dose administration of the hydromethanolic leaf extract and solvent fractions of *Podocarpus gracilis* in STZ-induced diabetic mice were observed in this study. The extract and fractions' ability to prevent muscle loss and tissue protein loss may be the cause of the rise in body weight [87, 88]. When blood glucose levels are poorly controlled in diabetics, lipid problems are frequently the main contributor to cardiovascular disease [89]. This study's STZ-induced diabetes results in elevated levels of triglycerides (TG), total cholesterol (TC), low-density lipoprotein (LDL), and decreased high-density lipoprotein (HDL) [90]. This discovery is completely consistent with the findings in cases of lipid abnormalities in diabetic individuals or diabetes animal models [91]. Administration of leaf extract and fractions of *Podocarpus gracilis* for two weeks significantly reduced serum STC, STG, VLDL-C, and LDL-C and increased HDL-C in a dose dependent manner. The active phytochemicals in *Podocarpus gracilis* extract and fractions (tannins, flavonoids, phenols, and triterpenes) can normalize cholesterol and act through several mechanisms including stimulating insulin secretion from pancreatic beta-cells, activating lipoprotein lipase, insulin-mediated lipolytic activity, or inhibiting lipogenic enzymes, may be responsible for this hypolipidemic activity [92].

## 5. Conclusion

This research found that in STZ-induced, normoglycemic, and oral glucose-loaded mice, the 80% hydromethanolic crude extract and fractions of *Podocarpus gracilis* significantly reduced blood glucose levels with weight gained. The results also revealed that the aqueous fraction has a low blood glucose reduction effect in streptozotocin-induced mice. The outcomes supported the theory that extracts' potential to free radical scavenging activity may also play a role in their anti-hyperglycemic effects. The findings provide encouraging proof of *Podocarpus gracilis'* effectiveness in treating diabetes and a justification for its continued use in traditional medicine.

## Figures and Tables

**Table 1:** Preliminary phytochemical screening of leaf extract and fractions.

Phytochemical	Crude extract	Aqueous fractions	Ethyl acetate	*n*-Hexane
Terpenoids	+	−	−	−
Alkaloids	+	−	+	+
Tannins	+	_	+	+
Flavonoids	+	+	+	+
Saponins	−	+	+	+
Phenols	**+**	+	+	+
Steroids	+	−	+	+
Glycosides	+	+	+	+
Anthraquinones	+	+	+	+

+ indicates presence and − denotes absent.

**Table 2:** The effect of leaf extracts of *Podocarpus gracilis* on BGL of normoglycemic mice.

Groups	Blood glucose level (mg/dl) in the different time intervals (hours)				
	0 H	1 H	2 H	3 H	4 H
DW10 ml	95.66±2.84	93.50±4.7	91.2±3.6	88.6±6.9	86.3±4.6
PGC100 mg	96.00±4.9	86±2.3	85.20±7.1	83.33±6.2	80.23±2.9
PGC200 mg	97.1±5.8	90.4±5.3	84.6±8.8^*∗∗∗*^^a^	76.2±5.5^*∗∗∗*^^a^^*∗∗*^	70.4±4.8^*∗∗∗*^^a^
PGC400 mg	98.3±7.5	94.23±3.6	87.3±4.3^*∗∗∗*^^a^	78.4±5.4	74.32±3.5^*∗∗∗*^^a^
GLC5mg	99.20±7.6	82.2±8.3	80.4±4.9^*∗∗∗*^^a^^*∗∗∗*^^b^	70.4±7.3^*∗∗∗*^^a^^*∗∗*^^b^	68.9±7.6^*∗∗∗*^^a^^*∗∗∗*^^b^

*n* = 6; PGC100 = *Podocarpus gracilis crude extract* 100 mg/kg, PGC200 = *Podocarpus gracilis* crude extract 200 mg/kg, PGC400 = *Podocarpus gracilis* crude extract 400 mg/kg, DW10 ml = distilled water 10 ml/kg, GLC5 = glibenclamide 5 mg/kg; ^a^compared with baseline blood glucose level (*t* = 0 h), ^b^compared with negative control group; ^*∗∗*^*p* < 0.01, ^*∗∗∗*^*p* < 0.001. ^a^indicates in all leaf extracts and fractions, phytochemicals like anthraquinones, glycosides, phenols, and flavonoids were identified. ^b^indicates in contrast, terpenoids were identified only in crude extract.

**Table 3:** The effects of the leaf extract of *Podocarpus gracilis* on oral glucose-loaded nondiabetic mice.

Groups	Blood glucose level (mg/dl) in the different time intervals (minutes)			
	0 min	30 min	60 min	120 min
DW10 ml	105.4±5.6	129.3±6.6^*∗∗∗*^^a^	128.3±6.0	125.3±4.2
PGC100 mg	107.4±6.2	130.3±5.5^*∗∗∗*^^a^	90.0±5.7^*∗∗∗*^^a^	80.1±8.0^*∗∗∗*^^a^
PGC200 mg	108.1±4.9	136.1±6.5^*∗∗∗*^^a^	88.1±5.4^*∗∗∗*^^a^	77.1±5.7^*∗∗∗*^^a^
PGC400 mg	109.4±6.0	134.3±7.6^*∗∗∗*^^a^	86.0±4.8^*∗∗∗*^^a^	75.3±3.8^*∗∗*^^a^
GLC5mg	106.2±5.5	133.2±7.7^*∗∗∗*^^a^	84.3±2.7^*∗∗∗*^^a^^*∗∗*^^b^	72.4±4.1^*∗∗*^^a^^*∗∗∗*^^b^

*n* = 6; PGC100 = *Podocarpus gracilis* crude extract 100 mg/kg, PGC200 = *Podocarpus gracilis* crude extract 200 mg/kg, PGC400 = *Podocarpus gracilis* crude extract 400 mg/kg, DW10 ml = distilled water 100 ml/kg, GLC5 = glibenclamide 5 mg/kg; ^a^compared with fasting blood glucose level (*t* = 0 h), ^b^compared with negative control group; ^*∗∗*^*p* < 0.01, ^*∗∗∗*^*p* < 0.001. ^a^indicates in all leaf extracts and fractions, phytochemicals like anthraquinones, glycosides, phenols, and flavonoids were identified. ^b^indicates in contrast, terpenoids were identified only in crude extract.

**Table 4:** Single dose antihyperglycemic effect of leaf extracts and fraction of *Podocarpus gracilis* in diabetic mice.

Groups	Blood glucose level (mg/dl) at different time interval (hours)				
	0 H	2 H	4 H	6 H	8 H
DC10 ml	314.4±2.3	316.2±9.3	318.0±7.1	317.22±8.4	319.00±4.2
CE100 mg	317±1.4	305.2±2.0	250.11±8.8	202.00±3.7^*∗∗*^^a^^*∗∗∗*^^b^	180±7.01^*∗∗*^^a^^*∗∗∗*^^b^
CE200 mg	318.4±6.1	287.4±3.4	220.06±6.8	195.8±5.6^*∗∗∗*^^a^^*∗∗∗*^^b^	177.60±7.6^*∗∗∗*^^a^^*∗∗∗*^^b^
CE400 mg	316.05 ± 4.5	280.3±2.8	210.5±5.1	185.4±4.8^*∗∗∗*^^a^^*∗∗∗*^^b^	174.2±6.2^*∗∗∗*^^a^^*∗∗∗*^^b^
EF100 mg	334.2±1.5	290.4±2.1	224.5±0.77	214.5±0.66^*∗∗*^^a^^*∗∗*^^b^	189.2±0.44^*∗∗*^^a^^*∗∗*^^b^
EF200 mg	326.5±1.1	286.3±2.1^*∗∗∗*^^b^	234.1±0.77^*∗∗∗*^^a^^*∗∗∗*^^b^	203.9±0.33^*∗∗∗*^^a^^*∗∗∗*^^b^	183.5±0.78^*∗∗∗*^^a^^*∗∗∗*^^b^
EF 400 mg	329.1±2.3	287.4±1.7^*∗∗∗*^^b^	238.5±1.3^*∗∗∗*^^a^^*∗∗∗*^^b^	180.4±0.54^*∗∗∗*^^a^^*∗∗∗*^^b^	174.3±0.71^a^^*∗∗∗*^^b^
AF100 mg	322.1±4.2	320.4±1.6	312.3±1.7	292.9±3.2	283.1±2.3
AF200 mg	320.2±7.3	317.3±6.3	313.4±7.7	291.4±5.6	277.4±3.1
AF 400 mg	318.3±1.5	315.6±5.3^*∗∗∗*^^b^	308.1±5.6^*∗∗∗*^^a^^*∗∗∗*^^b^	287.2±2.2^*∗∗∗*^^a^^*∗∗∗*^^b^	272.11±3.6^*∗∗∗*^^a^^*∗∗∗*^^b^
HE100 mg	328.4±1.3	298.5±5.3	232.1±6.6	221.4±7.5^*∗∗*^^a^^*∗∗*^^b^	198.1±1.7^*∗∗*^^a^^*∗∗*^^b^
HE200 mg	318.3±6.3	279.2±7.4^*∗∗∗*^^b^	243.1±7.7^*∗∗∗*^^a^^*∗∗∗*^^b^	208.1±2.6^*∗∗∗*^^a^^*∗∗∗*^	188.3±6.7^*∗∗∗*^^a^^*∗∗∗*^^b^
HE400 mg	321.5±3.2	284.1±4.9^*∗∗∗*^^b^	248.1±6.2^*∗∗∗*^^a^^*∗∗∗*^^b^	188.5±2.6^*∗∗∗*^^a^^*∗∗∗*^^b^	170.1±3.5^*∗∗∗*^^a^^*∗∗∗*^^b^
GLC5mg	319.04±8.4	275.6±8.3^*∗*^^a^^*∗∗*^^b^	201.001±5.5^*∗∗∗*^^a^^*∗∗∗*^^b^	180.5±7.3^*∗∗∗*^^a^^*∗∗∗*^^b^	168.02±7.9^*∗∗∗*^^a^^*∗∗∗*^^b^

Values are expressed as mean ± SEM (*n* = 6 mice in each group). *a* = compared to diabetes control; *b* = compared to baseline blood glucose level; CE = crude extract; AF = aqueous fraction; HE = hexane fraction; EF = ethyl acetate fraction; DC = diabetes control; GLC = glibenclamide. ^*∗*^ =*p* < 0.05, ^*∗∗*^ =*p* < 0.01, and ^*∗∗∗*^ =*p* < 0.001. ^a^indicates in all leaf extracts and fractions, phytochemicals like anthraquinones, glycosides, phenols, and flavonoids were identified. ^b^indicates in contrast, terpenoids were identified only in crude extract.

**Table 5:** The effect of repeated-dose antihyperglycemic of leaf extracts and fractions of *Podocarpus gracilis* in diabetic mice.

Groups	Blood glucose level in mg/dl (% reduction)		
	Day 0	Day 7	Day 14
NC DW10 ml	83.1±3.77	84.12±5.9^*∗∗∗*^^a^	85.13±6.5^*∗∗∗*^^a^
DC DW10 ml	314.4±2.3	317.4±2.8	320.42±1.7^*∗∗∗*^^b^
CE100 mg	317±1.4	195.34±4.5^*∗*^^a^^*∗*^^c^^*∗∗∗*^^b^	183.9±6.8^*∗∗*^^a^^*∗∗∗*^^b^^*∗∗∗*^^c^
CE200 mg	318.4 ±6.1	202.3±2.5^*∗∗*^^a^^*∗∗∗*^^b^^*∗∗∗*^^c^	179.2±1.45^*∗∗∗*^^a^^*∗∗∗*^^b^^*∗∗∗*^^c^
CE400 mg	316.05±4.5	191.21±3.6^*∗∗∗*^^a^^*∗∗∗*^^b^^*∗∗∗*^^c^	174.1±2.8^*∗∗∗*^^a^^*∗∗∗*^^b^^*∗∗∗*^^c^
EF100 mg	334.44±3.4^*∗∗∗*^^b^	201.55±3.66	190.3±6.1
EF200 mg	326.6±4.6^*∗∗∗*^^b^	210.3±3.1^*∗∗*^^a^^*∗∗∗*^^b^^*∗∗∗*^^c^	183.1±3.2^*∗∗∗*^^a^^*∗∗∗*^^b^^*∗∗∗*^^c^
EF400 mg	329.2±4.3^*∗∗∗*^^b^	193.2±2.7^*∗∗∗*^^a^^*∗∗∗*^^b^^*∗∗∗*^^c^	179.1±3.9^*∗∗∗*^^a^^*∗∗∗*^^b^^*∗∗∗*^^c^
HE100 mg	328.7±1.2	191.9±4.5	179.3±4.6
HE 200 mg	318.8±3.9	198.7±2.3^*∗∗*^^a^^*∗∗∗*^^b^	175.5±1.5^*∗∗∗*^^a^^*∗∗∗*^^b^
HE400 mg	321.5±3.3	187.2±4.4^*∗∗∗*^^a^^*∗∗∗*^^b^^*∗∗∗*^^c^	170.2±7.1^*∗∗∗*^^a^^*∗∗∗*^^b^
GLC5mg	319.04±8.4	182±8.8^*∗∗∗*^^a^^*∗∗∗*^^b^	165.5±5.00^*∗∗∗*^^a^^*∗∗∗*^^b^

Values are expressed as mean ± SEM (*n* = 6 mice in each group). *a* = compared to diabetes control; *b* = compared to baseline blood glucose level; *C* = compared to normal control; CE = crude extract; HE = hexane fraction; EF = ethyl acetate fraction; DC = diabetes control; GLC = glibenclamide. ^*∗*^ =*p* < 0.05, ^*∗∗*^ =*p* < 0.01, and ^*∗∗∗*^ =*p* < 0.001. ^a^indicates in all leaf extracts and fractions, phytochemicals like anthraquinones, glycosides, phenols, and flavonoids were identified. ^b^indicates in contrast, terpenoids were identified only in crude extract.

**Table 6:** The effects of leaf extract and fractions of *Podocarpus gracilis* on the body weight of diabetic mice.

Groups	Body weight of mice (gram)		
	Day 0	Day 7	Day14
NC DW10 ml/kg	25.21±1.4	26.01±6.2	26.55±4.4
DC DW10 ml/kg	30.21±7.5	25.3±3.4^*∗∗∗*^^b^	22.1±3.3^*∗∗∗*^^b^
CE100 mg/kg	25.45±2.2	25.54±7.9	26.60±7.2^*∗∗∗*^^a^
CE200 mg/kg	26.34±5.2	27.38±3.9^*∗∗∗*^^a^	29.14±7.3^*∗∗∗*^^a^
CE400 mg/kg	26.1±6.7	28.31±7.6^*∗∗∗*^^a^	30.34±5.1^*∗∗∗*^^a^
EF100 mg	23.1±0.13	24.2±0.44^*∗∗∗*^^a^	25.11±0.44^*∗∗∗*^^a^
EF200 mg	26.3±1.55	27.44±0.56^*∗∗∗*^^a^	28.7±0.33^*∗∗∗*^^a^
EF 400 mg	24.12±0.33	26.11±0.88^*∗∗∗*^^a^	27.3±080^*∗∗∗*^^a^
HE100 mg	22.11±7.4	22.91±2.0^*∗∗∗*^^a^	23.66±7.2^*∗∗∗*^^a^
HE 200 mg	23.4±0.22	24.6±0.21^*∗∗∗*^^a^	26.11±0.55^*∗∗∗*^^a^
HE400 mg	23.5±0.77	25.11±0.15^*∗∗∗*^^a^	27.4±0.23^*∗∗∗*^^a^
GLC 5 mg/kg	26.44±2.3	29.11±3.5^*∗∗*^^a^	31.5±2.4^*∗∗∗*^^a^

Values are expressed as mean ± SEM (*n* = 6 mice in each group). *a* = compared to diabetes control; *b* = compared to baseline body weight; CE = crude extract; HE = hexane fraction; EF = ethyl acetate fraction; DC = diabetes control; DW = distilled water GLC = glibenclamide. ^*∗*^ =*p* < 0.05, ^*∗∗*^ =*p* < 0.01, and ^*∗∗∗*^ =*p* < 0.001. ^a^indicates in all leaf extracts and fractions, phytochemicals like anthraquinones, glycosides, phenols, and flavonoids were identified. ^b^indicates in contrast, terpenoids were identified only in crude extract.

**Table 7:** The effects of leaf extract and fractions of *Podocarpus gracilis* on the lipid profile of diabetic mice.

Groups	STC (mg/dl)		TG (mg/dl)	LDL (mg/dl)	VLDL (mg/dl)	HDL (mg/dl)
NC10 ml	86.01 ± 1.4^*∗∗∗*^		77.4 ± 1.3^*∗∗∗*^^a^	32.4 ± 1.5^*∗∗∗*^^a^	18.2.1 ± 7.3^*∗∗∗*^^a^	35.5 ± 7.8^*∗∗∗*^^a^
DCW10 ml	178.4 ± 5.2^*∗∗∗*^^b^		173.5 ± 5.4^*∗∗∗*^^b^	127.7 ± 4.2^*∗∗∗*^^b^	30.31 ± 5.1^*∗∗∗*^^b^	20.4 ± 1.3^*∗∗∗*^^b^
CE00 mg	167.3 ± 6.5^*∗∗*^^a^		147.3 ± 8.2	109.5 ± 6.6	27.3 ± 2.2^*∗∗∗*^^a^^*∗∗∗*^^b^	30.3 ± 5.1
CE200 mg	161.33 ± 3.2^a^		140.3 ± 1.1^*∗∗∗*^^a^^*∗∗∗*^^b^	102.3 ± 3.0^*∗∗∗*^^b^	24.88 ± 7.6^*∗∗∗*^^b^	31.22 ± 4.1^*∗∗∗*^^a^
CE400 mg	159.44 ± 6.7^*∗∗∗*^^a^^*∗∗∗*^^b^		138.38 ± 5.3^*∗∗∗*^^a^^*∗∗∗*^^b^	100.3 ± 3.2^*∗∗∗*^^a^^*∗∗∗*^^b^	23.21 ± 6.4^*∗∗∗*^^a^^*∗∗∗*^^b^	33.5 ± 8.1^*∗∗∗*^^a^
EF100 mg	168.3 ± 3.5^*∗∗*^^a^^*∗∗*^		148.9 ± 2.8^*∗∗∗*^^a^^*∗∗∗*^^b^	110.3 ± 0.77^*∗∗∗*^^a^^*∗∗∗*^^b^	27.6 ± 2.4^*∗∗∗*^^a^^*∗∗∗*^^b^	29.9 ± 0.65^*∗∗∗*^^a^^*∗∗∗*^^b^
EF200 mg	163.7 ± 1.2^*∗∗∗*^^a^^*∗∗∗*^^b^		140.1 ± 2.7^*∗∗∗*^^a^^*∗∗∗*^^b^	103.5 ± 0.44^*∗∗∗*^^a^^*∗∗∗*^^b^	24.55 ± 0.43^*∗∗∗*^^a^^*∗∗∗*^^b^	31.3 ± 2.5^*∗∗∗*^^a^
EF400 mg		161.4 ± 2.7^*∗∗∗*^^a^^*∗∗∗*^^b^	140.32 ± 1.43^*∗∗∗*^^a^^*∗∗∗*^^b^	100.23 ± 1.6^*∗∗∗*^^a^^*∗∗∗*^^b^	23.97 ± 0.21^*∗∗∗*^^a^^*∗∗∗*^^b^	35.1 ± 0.45^*∗∗∗*^^a^
HE100 mg		166.3 ± 24^*∗∗*^^a^^*∗∗∗*^^b^	146.6 ± 2.4^*∗∗∗*^^a^^*∗∗∗*^^b^	108.3 ± 0.55^*∗∗∗*^^a^^*∗∗∗*^^b^	26.3 ± 0.33^*∗∗∗*^^a^^*∗∗∗*^^b^	29.33 ± 0.5^*∗∗∗*^^a^^*∗∗∗*^^b^
HE200 mg		160.4 ± 3.1^*∗∗∗*^^a^^*∗∗∗*^^b^	139.3 ± 0.44^*∗∗∗*^^a^^*∗∗∗*^^b^	101.4 ± 0.1^*∗∗∗*^^a^^*∗∗∗*^^b^	24.11 ± 0.99^*∗∗∗*^^a^^*∗∗∗*^^b^	30.5 ± 2.4^*∗∗∗*^^a^
HE 400 mg		158.3 ± 6.6^*∗∗∗*^^a^^*∗∗∗*^^b^	137.5 ± 0.8^*∗∗∗*^^a^^*∗∗∗*^^b^	100.1 ± 2.2^*∗∗∗*^^a^^*∗∗∗*^^b^	22.2 ± 0.7^*∗∗∗*^^a^^*∗∗∗*^^b^	36.01 ± 0.22^*∗∗∗*^^a^
GLC 5 mg	96.44 ± 2.77^*∗∗∗*^^a^		83.12 ± 4.7^*∗∗∗*^^a^^*∗∗∗*^^b^	37.1 ± 5.5^*∗∗∗*^^a^	17.33 ± 9.1^*∗∗∗*^^a^	36.61 ± 4.4^*∗∗∗*^^a^

Values are expressed as mean ± SEM (*n* = 6 mice in each group). *a* = compared with diabetic control, *b* = compared with normal control group, GLC = glibenclamide, DC = diabetic control, DW = distilled water, NC = normal control, STC = serum total cholesterol, TG = Triglyceride, HDL-c = high-density lipoprotein cholesterol, VLDL-c = very-low-density lipoprotein cholesterol, LDL-c = low-density lipoprotein cholesterol, *CE* = crude extract; *n*-HE = hexane fraction; EF = Ethyl acetate fraction; DW = distilled water.^*∗*^ = *p* < 0.05, ^*∗∗*^ = *p* < 0.01, ^*∗∗∗*^ = *p* < 0.001. ^a^indicates in all leaf extracts and fractions, phytochemicals like anthraquinones, glycosides, phenols, and flavonoids were identified. ^b^indicates in contrast, terpenoids were identified only in crude extract.

**Table 8:** Antioxidant activities of the leaf extract of *Podocarpus gracilis* in diphenyl-2-picrylhydrazyl assay.

Concentrations (*μ*g/ml)	% inhibition of DPPH	
	Leaf extract	Ascorbic acid
62.5	3.5 ± 0.21	11.23 ± 0.44
125	3.9 ± 0.34	25.01 ± 0.05
250	8.1 ± 0.39	45.51 ± 0.31
500	17.4 ± 0.61	53.54 ± 0.37
1000	27.1 ± 0.41	66.6 ± 0.24
2000	43.13 ± 0.33	80.15 ± 0.12
IC50	8.2 ± 0.31	3.3 ± 0.66

The IC_50_ value denotes the concentration of the sample required to scavenge 50% DPPH radicals.

## Data Availability

The corresponding author can provide the datasets that were used and/or analyzed in this study upon reasonable request.

## Appendix

The procedures for the leaf extract and fractions of *Podocarpus gracilis* phytochemical screening are as follows:

1. Saponins test: to 0.5 gm of crude and fractions, 2 ml of distilled H_2_O was added. After shaking vigorously, a stable foam was observed for 10 minutes. A stable persistent foam was observed that indicated the presence of saponins.
2. Terpenoids test: to 0.25 gm of crude and fractions, chloroform was added. Then, a layer was created after 3 ml concentrated H_2_SO_4_ was added. At the interface, the appearance of reddish-brown color indicated the presence of terpenoids.
3. Test for flavonoids: to 0.2 gm of the crude and fractions, 5 ml 95% ethanol, few drops of concentrated HCL, and 5 gm of magnesium turnings were added. The pink color appearance indicated presence of flavonoids.
4. Test for glycosides: to 2 ml of the extract, 2 ml of glacial acetic acid, FeCl_3_, and concentrated H_2_SO_4_ was added. At the junction of two liquids, reddish brown color and the bluish color at the upper layer were formed, which indicated the presence of glycosides.
5. Test for alkaloids: after a mixture of 2-3 drops of the crude extract and fraction and 1 ml of 1% HCl was heated, few drops of Wagner's reagent was added to the mixture. A precipitate was observed, which is taken as a positive test for presence of alkaloids.
6. Anthraquinones test: 100 mg of the sample was vigorously shaken with benzene (10 ml) and then filtered. To the filtrate, 5 ml of 10% ammonia solution was added. The formation of red pink color showed an indication for the presence of anthraquinones.
7. Test for tannins: 1% gelatin solution having sodium chloride was added to the crude extract and fraction. The white precipitate indicates the presence of tannins.
8. Test for steroids: to 2 ml of the extract 1 ml of chloroform, 1 ml of H_2_SO_4_ was added. Then, after shaking, the shaking causes the acid layer to turn yellow with a green florescence, and the chloroform layer to turn red. This color appearance was an indication of the presence of steroids.
9. Test for phenols: 2 ml of 2% FeCl_3_ solution was mixed with 2 ml of the extract. Appearance of bluish black color was a positive indication for the presence of phenols.

## Abbreviations

IDF: International Diabetes Federation
ANOVA: Analysis of variance
BGL: Blood glucose level
DPPH: Diphenyl-1-picrylhydrazyl
DM: Diabetes mellitus
FPG: Fasting plasma glucose
OGTT: Oral glucose tolerance test.
